# Incorporation of Nurse Initiated Management of Antiretroviral Treatment course within the undergraduate nursing programme North West province

**DOI:** 10.4102/hsag.v28i0.2286

**Published:** 2023-10-13

**Authors:** Kabelo S. Bonokoane, Jeanettte M. Sebaeng, Seepaneng S. Moloko-Phiri

**Affiliations:** 1 Department of Nursing, Faculty of Health Sciences, North-West University, Mahikeng, South Africa; 2 Department of Nursing, Faculty of Health Sciences, University of the Free State, Bloemfontein, South Africa

**Keywords:** course, incorporation, NIMART, nurse educator, programme

## Abstract

**Background:**

According to the 90-90-90 strategy, the focus is on 90% of people living with HIV and/or AIDS knowing their HIV status, initiated on antiretroviral treatment and achieving viral suppression. The challenge is that only 74% of people living with HIV and/or AIDS are on antiretroviral treatment, and HIV mortality still occurs. Literature recommends the incorporation of a Nurse Initiated Management of Antiretroviral Treatment (NIMART) course within the undergraduate nursing programme to capacitate new nurses to manage people living with HIV and/or AIDS immediately after completion of their training. However, the NIMART course is still not incorporated, and there is dearth of information on this topic in North West Province (NWP).

**Aim:**

To explore and describe nurse educators’ perceptions regarding the incorporation of NIMART course within the undergraduate nursing programme in NWP.

**Setting:**

The setting of this research study was nursing education institutions of the NWP.

**Methods:**

Phenomenography qualitative research design was followed. Twelve nurse educators underwent purposive selection and unstructured individual interviews were conducted. The research co-coder verified the findings. There were ethical considerations and trustworthiness maintained throughout the study.

**Results:**

Main themes that emerged in this study depicted benefits and challenges associated with NIMART course incorporation within the undergraduate nursing programme as stated in Table 1.

**Conclusion:**

This study concluded that NIMART course incorporation within the undergraduate nursing programme is a good and relevant idea, which requires human and non-human resources.

**Contribution:**

The study contributed new knowledge on how nurse educators perceive the NIMART course incorporation within the undergraduate nursing programme in NWP.

## Introduction

Human immunodeficiency virus (HIV) is still the deadliest infection among human beings to date, despite the use of Antiretroviral treatment (ART) (Cunha et al. 2021:1; WHO 2022). According to the World Health Organization’s (WHO 2022) global summary report, there were 38.4 million People Living with HIV and/or AIDS (PLWHA), 1.5 million new HIV infections and 65 000 deaths by the end of 2021. In an attempt to minimise the infection rates and control mortality from HIV, the Joint United Nations Programme on HIV and/or AIDS (UNAIDS) introduced the 90-90-90 strategy during the 20th International AIDS Conference held in Australia (UNAIDS 2017:8). The strategy states that by 2020, 90% of PLWHA should know their status, 90% of PLWHA initiated on ART and 90% of people on ART virally suppressed (UNAIDS 2017:8). According to O’Byrne, without more healthcare workers to increase access to HIV healthcare services, healthcare facilities will remain strained (O’Byrne et al. 2021:7). Before the UNAIDS strategy, there was a shortage of four million healthcare workers in sub-Saharan Africa, Asia and America (WHO 2008:6).

To decentralise HIV healthcare services among PLWHA, South Africa (SA) adopted the WHO’s 2008 Task Shifting Strategy and introduced Nurse Initiated Management of ART (NIMART) course to increase ART coverage (NDoH 2016:6). The introduction of the NIMART course was to include nurses in the management of PLWHA, as this task has been doctor-driven since 2004 (NDoH 2016:6). There has been great support for Task Shifting as a key strategy to improve access to HIV care services (O’Byrne et al. 2021:7). Furthermore, O’Byrne et al. (2021:7) emphasised Task Shifting from doctors to nurses as a feasible way to increase access to HIV services, such as Pre-Exposure Prophylaxis (PrEP) to high-risk populations. Despite the introduction of the Task Shifting Strategy, South Africa remains the epicentre for HIV, in which there is a serious gap in initiating all PLWHA on ART (UNAIDS 2021:2). Data collected from both the private and public sectors indicate that as of March 2021, South Africa was at 93-73-88 in terms of performance against the UNAIDS 90-90-90 target across its population (SANAC 2020–2021:7). Regardless of SA having the largest ART programme globally, much work still needs to be done to reach the second 90 target.

Since the inception of the NIMART course in South Africa, it is only for Professional Nurses (PN) registered with the South African Nursing Council (SANC) (NDoH 2016:9). Mngqibisa et al. (2017:156) maintain that NIMART can be effective when offered to student nurses during training in undergraduate programmes to curb human resource constraints at facilities and increase ART access. This is further emphasised by Warren et al. (2018:119), who argue that failure to integrate NIMART into the undergraduate nursing programme may leave students with a lack of knowledge in basic HIV screening, prevention and management of patients. Furthermore, student nurses reportedly lacked the knowledge and skills to manage PLWHA on completion of the undergraduate programme (Modeste & Adejumo 2015:346). Conversely, the study conducted in KwaZulu-Natal affirmed the overall improvement of knowledge in all HIV aspects when student nurses and PNs undertake the NIMART course (Mngqibisa et al. 2017:156). The recommendation is for the incorporation of the NIMART course within the undergraduate nursing programmes following its positive benefits (Lekhuleni, Kgole & Mbombi 2015:1; Zuber et al. 2014:520).

Several Nursing Education Institutions (NEI) in Africa have been at various stages of planning a nation-wide sustainable curriculum for nursing and midwifery programmes (Nyoni & Botma 2020:6). However, for the past 10 years, nursing and midwifery curriculum in South Africa still excludes information on NIMART. Moreover, there is a dearth of literature in North West Province (NWP) regarding incorporation of the NIMART course within the undergraduate nursing programme in NWP. In SA, nurse educators are responsible for designing and reviewing the nursing curriculum based on the programme outcomes for undergraduate nursing programmes as outlined by SANC (2014:2). Therefore, this study explored and described the perceptions of nurse educators regarding incorporation of the NIMART course within the undergraduate nursing programme in NWP, South Africa.

## Research methods and design

The study utilised qualitative, phenomenography research design. Phenomenography is a qualitative research design that explores different ways people perceive, experience, conceptualise and understand phenomenon in their world by using descriptive data (Rands & Gansemer-Topf 2016:1). Phenomenography research design was appropriate in this study as it allowed the researcher to explore and describe NE perceptions regarding the incorporation of the NIMART course within the undergraduate nursing programme.

### Study setting

The study was conducted at two Nursing Education Institutions (NEIs) (university and college) in NWP, SA, which offers nursing education and training. The NEIs referred to in this study have two sub-campuses each. Two campuses are in Ngaka Modiri Molema district and two are in Dr Kenneth Kaunda district. The two NEIs are 179 km apart. One principal and two campus heads manage the two colleges, while the university has school directors in each campus. These NEI’s were selected because that is where NEs are based and also form part in the curriculum development.

### Population and sampling

The population for this study was 43 NEs from NEIs in NWP:

This study utilised non-probability sampling, which is a type of sampling that uses human judgement to make some members of the population more likely for selection than are others (Bryman & Bell 2017:380). Twelve NEs were purposively selected at the respective NEIs. The criteria used for inclusion in the study comprised nurse educators with teaching responsibilities in the undergraduate nursing programme, as well as those registered with SANC.

Preceptors and clinical accompanists were excluded as they spend less time with students and do not often form part of the undergraduate curriculum committee members within the NEIs.

### Data collection

In this study, unstructured individual interviews were conducted using one central open-ended research question and probing questions. Probing was for clarity seeking and for stimulating participants for more information. The interviews were conducted through Microsoft Teams application and thereafter transcribed. According to Menzies, Williams and Zimmermann (2016:125), an interview guide refers to a list of questions the researcher uses during an interview. The following question was asked:

- What are your perceptions regarding incorporation of Nurse Initiated and Managed Antiretroviral Treatment course in the undergraduate nursing programme in NWP?

### Data analysis

Thematic data analysis was used to analyse the data. Thematic data analysis involves detecting themes and patterns, which emerge from participants and are central to credible research, because it is a flexible method for data analysis in qualitative research; its application can also be in other kinds of learning (Maguire & Delahunt 2017:1).

Maguire and Delahunt’s (2017:4) six-phase guide to conduct thematic data analysis, which is a useful framework for conducting data analysis, is as follows:

#### Step 1: The researcher firstly becomes familiar with data

This step was achieved through repeated reading of the transcribed, verbatim data about perceptions of NEs regarding incorporation of the NIMART course within the undergraduate nursing programme to get a sense of the information provided, thus becoming familiar.

#### Step 2: The researcher generated initial codes of data

The researcher reduced the transcribed data into small sections, which focused on answering the research question.

#### Step 3: The researcher searched for themes

The researcher searched for patterns of data, which captured something interesting and significant about the research question. Afterwards, the researcher examined the codes generated in step 2 and fit them together into themes.

#### Step 4: Review of themes

The researcher reviewed, modified and developed primary themes from those searched in step 3 to check whether they made sense and whether the transcribed data supported them. At this stage, the researcher gathered the data relevant to each theme to verify its support.

#### Step 5: The researcher defined themes

*The researcher* did final refinements and developed sub-themes that were still available, including how these sub-themes related to the main themes.

#### Step 6: The researcher wrote the findings

Thematic data analysis was utilised to write-up findings, which answered the research question.

### Ethical considerations

The North-West University Health Research Ethics Committee (NWU-HREC) granted ethical approval for this study (ethics number: NWU-00297-21-S1). Respect for persons, beneficence, justice was maintained. Also informed consent was obtained from the participants prior to data collection in line with *POPIA Act*. Participants were made aware that participating was voluntary and that they could withdraw from participating anytime without explaining themselves.

## Results

The results of the study are presented in themes and sub-themes that resulted from the qualitative data collected among 12 participants. All 12 participants had more than 5 years of experience facilitating undergraduate nursing programme curriculum and were permanent employees of their designated NEI. Table 1 illustrates the four main themes and their sub-themes that emerged in this study.

**TABLE 1:** Themes and sub-themes.

Theme	Sub-themes
1. Incorporation of NIMART course into the undergraduate programme is a good idea and initiative	1.1 Benefits to student nurses 1.2 Benefits to community 1.3 Benefits to Nursing Education Institutions
2. NIMART course to be a module on its own	2.1 NIMART course to be recognised as a module 2.2 Stakeholder collaboration to recognise NIMART course as a speciality
3. Challenges experienced without the NIMART course in the undergraduate nursing programme	3.1 Knowledge gap 3.2 Poor patient management 3.3 Cost of training nurses on completion of undergraduate programme
4. Challenges that might hinder NIMART course incorporation into the undergraduate nursing programme	4.1 Curriculum overload

NIMART, Nurse Initiated Management of Antiretroviral Treatment.

### Theme 1: Incorporation of Nurse Initiated Management of Antiretroviral Treatment course into the undergraduate programme is a good idea and initiative

This is the first theme that emerged from this study and it highlighted the importance of incorporating NIMART into the undergraduate nursing programme. The following sub-themes emerged, benefits to the students, community and NEI.

#### Sub-theme: 1.1 Benefits to student nurses

In this study, the majority of participants affirmed that incorporation of the NIMART course would allow students to acquire more knowledge, skills and confidence to manage People Living with HIV and/or AIDS (PLWHA). The participants expressed the following:

‘The students will be competent in NIMART and when are competent it will increase their confidence in managing people with HIV or those who need NIMART services so competency and confidence of the students.’ (Participant C)

Another participant said:

‘If you are NIMART trained before you even go to the clinics or before you go to practise I think you will have a better understanding and you will be able to work more sufficiently in practise.’ (Participant E)

To further support that the NIMART course benefits students, another participant expressed:

‘If somebody is empowered they are confident in what they are doing because they know exactly what they have been taught. They are knowledgeable so if you are knowledgeable you got all the information you got the skill then you are confident and then you can deliver quality care to the patient.’ (Participant J)

Students would have clinical competency in managing PLWHA, and this was echoed as follows:

‘When they go to practise, they will be able to take the history comprehensively whereby again they will be able to use WHO staging criteria … they will be able to assess aah opportunistic infections aah manage them in time … management of STI’s at an early stage is very important and early diagnosis and management of opportunistic infections is very important. students will be knowledgeable will start initiation of adults and children because … acquire skills necessarily to monitor patients from NIMART including the management of kidney failure, they will acquire skills from counselling and adherence of clients on NIMART again they will be knowledgeable on a pre and post counselling. They will also be knowledgeable on the monitoring of clients including children, monitor for the viral loads and checking the CD4 count.’ (Participant H)

‘I think it’s very important if I was a student and it was part of the course, I would have much large understanding of ARV and how it works.’ (Participant E)

Other participants mentioned that when undergraduate nursing students complete the NIMART integrated training they will share the information learned with professional nurses already in practice:

‘So they will be able to share that knowledge with other nurses after they have been trained on management and treatment of HIV, STI and Tuberculosis.’ (Participant B)

#### Sub-theme 1.2: Benefits to community

Several participants in this study indicated that the community would benefit with the NIMART course incorporated into the undergraduate nursing programme through the supermarket approach, whereby everything is one stop shop, meaning all patients complaints would be treated in one consulting room thus saving time:

‘Because when the clients go to the facilities remember with the primary healthcare approach it is that it should be a supermarket approach then it means that when a client enters a consulting room this client will receive everything that needs to be on him and on his way out as he leaves the consulting room everything will be done to this client.’ (Participant I)

There would be no need for patients to be referred to other facilities and travel long distances to access HIV services, as transport issues would be solved as people do not have money to be travelling around. This was explained as follows:

‘Everything will be one stop shopping you are coming for your hypertension medication then professional nurses trained in NIMART would be able to take care of you for your HIV treatment regime and also it means it will save the society like the community a lot of money, time and what “yeah” all that’ Community will benefit in the sense that whenever the nurse is placed, then we know that the nurse will holistically be able to take care of the patient because we have challenges of patients having to travel long distance to access health care that is still a challenge.’ (Participant L)

Another participant explained the benefits to the community as follows:

‘There will be no need to refer them to someone else they can be able to help the patients there, … some of the patients are not on treatment because they have so many challenges around that fact you say to the patients okay go to such clinic or come back on such a date so that you can be seen by someone who is been trained for this specific course and then the patients doesn’t pitch up based on so many challenges. One of it being maybe they don’t have transport to come to the clinic for that so if one is a professional nurse trained in NIMART the patient can get that treatment right at that first consultation.’ (Participant G)

‘It will mean that now our whole community will receive treatment in time they won’t just go disappear without any type of trace because … they experience challenges one of it being financial challenges maybe the patient have to catch a taxi to go to the clinic now they are there you telling them to come back again they don’t have money for that.’ (Participant G)

Community mortality statistics will reduce, as patients will be started on treatment early, thereby reducing Loss to Follow-Up (LTFU) rate exacerbated by patients’ financial challenges to reach the facilities offering NIMART services:

‘Obviously the patients will be started early on the treatment by the people who know and just reducing the stats and mortality … of people living with HIV.’ (Participant F)

#### Sub-theme 1.3: Benefits to Nursing Education Institutions

In this study, some participants indicated that the NEIs would benefit from positive feedback from the community regarding the NIMART services rendered by new graduate nurses trained in NIMART:

‘Our community, our people, our patients will come back and say we really thank the institution for the people they trained who are well cooked and have relevant qualifications and experience when coming to NIMART … it will benefit a lot of people it will benefit the students, institution, community, patients their families and so on.’ (Participant C)

Another benefit was that students who receive NIMART incorporated training would have a market advantage compared to those not trained, thereby putting the NEI on the map:

‘If this kind of students comes out with that competency, it means the student themselves will be marketable, it puts our institution into the map again without being really selfish.’ (Participant K)

Furthermore, participants expressed their readiness and interest to incorporate the NIMART course should the NEI endorse such an innovation:

I am saying when we revise it, like this 3-year Diploma one when we revise it, we are definitely going to do this, it will be good if we incorporated it, we will incorporate it.’ (Participant F)

‘I agree 100% that NIMART must be incorporated.’ (Participant C)

### Theme 2: Nurse Initiated Management of Antiretroviral Treatment course to be a module on its own

The participants recommended that NIMART course be a module on its own, and the following sub-themes emerged:

#### Sub-theme 2.1: Nurse Initiated Management of Antiretroviral Treatment course to be recognised as a module

In this study, some participants explained there was a need to recognise HIV and AIDS as a killer that is harmful to society. Therefore, when incorporated into the undergraduate programme, the NIMART course should be a module on its own with its own National Qualifications Framework (NQF) level, module notional hours, module credits and not encapsulated within modules such as general nursing science or community nursing:

‘I am saying it should be a module on its own its should not be put under other module like let us say general nursing science or community nursing science it must be on its own with its own NQF level.’ (Participant C)

‘I am suggesting that it should have 12 credits and we know that 12 credits need at least 120 notional hours so this will be a full module were students will be given theory and in class given assignments, texts, examinations.’ (Participant C)

#### Sub-theme 2.2: Stakeholder collaboration to recognise Nurse Initiated Management of Antiretroviral Treatment course as a speciality

Participants indicated the important need for stakeholder collaboration to take HIV and AIDS management in cognisance and regard it as a speciality as it is a public health concern:

‘I say collaboration for an example SANC as a regulatory body can also take into consideration the HIV & AIDS management together the Department of Higher Education and Training (DHET), Department of Health (DoH), South African Nursing Council (SANC) and nursing education institutions. They should come together to recognise NIMART course as a speciality … because it’s a public health crisis.’ (Participant D)

### Theme 3: Challenges experienced without the Nurse Initiated Management of Antiretroviral Treatment course in the undergraduate nursing programme

In this study, a few challenges were highlighted and were explained in three subthemes.

Three sub-themes emerged as follows: knowledge gap regarding NIMART, poor patient management and cost of training nurses on completion of undergraduate programme.

#### Sub-theme 3.1: Knowledge gap

Participants in this study explained that it is fruitless for more student nurses to graduate in large numbers from different NEIs without being knowledgeable on NIMART. Often new graduate nurses lack the knowledge to manage PLWHA, and such incompetency contributes towards patient mismanagement:

‘Nursing students graduate from different institutions in large numbers and to me it is fruitless for them to graduate without more insight of proper management of patients living with HIV & AIDS.’ (Participant D)

‘If you are not NIMART trained you cannot initiate the patient …it means they will have to wait for the doctor just like when it was done previously which will waste patient time.’ (Participant A)

‘Books used within the institution of higher learning are old and lack NIMART information. My experience is that ey … NIMART trained nurse’s focus into the current HIV and/or AIDS management.’ (Participant K)

#### Sub-theme 3.2: Poor patient management

Participants indicated that if newly qualified nurses were not NIMART trained, there is a possibility they may lack the skills to manage PLWHA, particularly pregnant women. In addition, the delayed incorporation of the NIMART course has led to mortality among PLWHA.

The following participants said:

‘Most of professional nurses often manage pregnant HIV positive women poorly because they do not have proper knowledge on how to treat those patients.’ (Participant B)

‘Well the study on incorporation of NIMART course into undergraduate programme come late when a lot of people have already died because of not receiving care from people who have relevant experience and qualification but something can still be done we cannot just folds’ hands.’ (Participant C)

#### Sub-theme 3.3: Cost of training nurses on completion of undergraduate programme

It is expensive to offer the NIMART course post-undergraduate qualification, as accommodation and meals have to be booked for a week for participants to attend. These nurses have to attend the training during the clinic working time leading to staff shortages and poor service delivery:

‘If our students can be trained while they are still doing nursing, kere (am saying) it will be less expensive as compared to when they are trained while they are registered nurses. Number one this training takes a week if I am not mistaken and by that time accommodation and meals have to be booked for those professional nurses.’ (Participant B)

‘Okay because we have shortage of staff and when for example 2 professional nurses for that particular week are on NIMART training it means patients will not be attended too … mortality and mobility rates increase due to shortage of staff.’ (Participant B)

### Theme 4: Challenges that might hinder Nurse Initiated Management of Antiretroviral Treatment course incorporation into the undergraduate nursing programme

Participants shared few challenges that might hinder incorporation of NIMART course into undergraduate programme. The sub-themes below give emphasis to this main theme.

#### Sub-theme 4.1: Curriculum overload

Some participants in this study stated that the undergraduate nursing programme curriculum was overloaded:

‘This new programme, the R171 there is too much content so I don’t know how will they be able to just incorporate it … in first year these students are doing maybe 5 system if I am talking about the biological science within a limited time.’ (Participant A)

‘This R171 the new curriculum iyhooo! Is a headache on its own so maybe when we are already settled in terms of this new curriculum maybe yeah we can start introducing something else. The period has been cut off from 4 years to 3 years so accommodating something else I don’t think is going to be easy.’ (Participant G)

The following was in support of a loaded curriculum:

‘So it is a tedious process and I don’t think it will be something that will be done like tomorrow it will take a while given the fact that we are just starting with a new curriculum.’ (Participant I)

### Measures of trustworthiness

Trustworthiness refers to the effort to signal openness, relevance, thoughtfulness in data collection and analysis, epistemology and methodological correspondence, including the researcher’s self-understanding about the findings (Brink et al. 2018:110). Utilised was Lincoln and Guba’s framework of 1985, which explains application of credibility, transferability, dependability and confirmability to ascertain trustworthiness (Polit & Beck 2017). To establish credibility, the researcher applied prolonged engagement strategy (Polit & Beck 2017:986) and recorded the interview, through use of Microsoft Teams virtual application, to check for relevance and congruence in the data being transcribed, so that the data truly represents the participants’ voice. The achieving of dependability was by making transcribed data and virtual records containing participant’s voices available to the research supervisors, to verify and determine there was no data missing nor the researcher misguided and finally determine if the findings were acceptable and consistent. The researcher gave sufficient thick descriptions of descriptive data so that the consumer could determine if the study findings are applicable in their settings to ascertain transferability of the findings (Polit & Beck 2017:983). Confirmability was achieved by submitting transcribed information and virtual records for co-coding, as this assisted in the verification of findings and ascertained that the findings, conclusion and recommendations truly represented what the researcher transcribed.

## Discussion

### Nurse Initiated Management of Antiretroviral Treatment course benefits for student nurses, community and nursing education institutions

#### Student benefits

Participants in this study explained the NIMART course incorporation in the undergraduate nursing programme was a good idea and initiative, which would benefit student nurses, the community and NEIs. Findings are consistent with those of Ngcobo and Mchunu (2019:1), which states that employers, students and healthcare recipients may all benefit immensely from HIV and AIDS curriculum when offered during the pre-service training. Another important finding was that the NIMART course would also prepare students to manage PLWHA post completion of their undergraduate nursing programme. The research study by Warren et al. (2018:119) also found that the incorporation of the NIMART course within the undergraduate nursing programme benefits students, as they would have acquired knowledge and skills regarding HIV and AIDS. This study also emphasises that the incorporation is necessary because the HIV statistics among PLWHA is high, and HIV is seen in all aspects of nursing care whether psychiatry or midwifery and should be treated as a chronic disease. As such, the incorporation of the NIMART course is imperative in the curriculum to meet societal needs of the community and address shortage of NIMART nurses. Similarly, Mboweni and Makhado (2020:1) also mentioned the need to advance NIMART training and implementation through the standardisation of NIMART curriculum, introduction of pre-service NIMART training in institutions of higher learning and addressing staff shortages. In addition, the study of Rossini et al. (2021:659), on nursing curriculum, explained the need to pursue the design of flexible training paths that consider the needs of students and promotion of social activities. However, there is no NIMART information presented in the undergraduate nursing programme, and these needs recognising as a killer, which destroys society.

Participants indicated the NIMART course would benefit students in terms of knowledge, skills and confidence needed for them to render HIV and AIDS services among PLWHA. Among these benefits, participants reported students would be able to take comprehensive history, diagnose patients early, use the WHO’s clinical staging criteria, assess opportunistic infections, manage sexually transmitted infections (STI’s) at an early stage, manage kidney failure, monitor patients’ viral loads and CD4 count and be able to provide pre- and post-counselling. Ngcobo and Mchunu (2019:1), Lekhuleni et al. (2015:1) and Zuber et al. (2014:520) support the findings that incorporation of the NIMART course in the undergraduate programme increases knowledge, confidence, skills and positive attitudes of new graduate nurses in managing PLWHA. Additionally, findings revealed students would be able to share the knowledge acquired with the professional nurses who are already working at the facilities for improved service delivery. Ngcobo and Mchunu (2019:8) and Naidoo et al. (2017:9) support the finding that students who have undergone an HIV and AIDS programme are empowered and able to share such knowledge with PN who have never had exposure to such curriculum and are in the field. Furthermore, if the student nurses are NIMART trained, they will be prepared for practice in future post-graduation, thereby being able to work efficiently without supervision, even when placed alone in rural areas. However, Ngcobo and Mchunu (2019:7) indicated that in SA, the majority of nursing students graduate without essential HIV and AIDS training required for them to perform their work successfully after graduating.

### Community benefits

Participants in this study stated the NIMART course incorporation in the undergraduate nursing programme would benefit the community by addressing the challenges they encounter to access HIV and/or AIDS healthcare services. There may be some elimination of communities’ financial problems in that they could access treatment at their nearby facility without the need for them to transfer to facilities where there is someone with NIMART skill. New graduate nurses will offer holistic care by applying the supermarket approach whereby the treating of HIV and AIDS, including other chronic conditions, will be on the same day in one consulting room. This finding is supported by Mngqibisa et al. (2017:153) who stated that barriers such as poverty and the inability to travel long distances to access healthcare facilities, add to the critical need to decentralise HIV and AIDS care to primary healthcare settings; hence, NIMART incorporation in undergraduate programme is imperative to address these barriers. As patients will receive treatment in one consulting room, the waiting time at facilities will reduce. However, because of the shortage of nurses trained in the NIMART course, existing NIMART nurses state that, they feel overwhelmed with the increased workload, as the number of PLWHA is high at facilities and they have a lengthy wait before their turn to receive HIV services (Maronda 2021:72) and (Mboweni & Makhado 2020:7). Furthermore, the mortality rate and HIV statistic may reduce when there is incorporation of the NIMART course, as patients will receive their ART treatment early without encountering financial challenges perpetuated by catching a taxi to the clinic. The financial challenges have contributed to patients being Lost to Follow-Up (LTFU), as the clinic will send them home, with another date to return to the facility. Mboweni and Makhado (2020:7) support the findings of this study that effective NIMART training will facilitate improved health outcomes, of PLWHA by linking them on ART, retaining them on ART to reduce LTFU and relieve pressure on NIMART nurses, thereby reducing mortality and increasing life expectancy. In addition, Maronda (2021:2) highlighted the need for community outreach teams to reach areas far from the clinics to reduce transportation costs for the patients and the number of patients being LTFU.

### Nursing education institution benefits

Participants reported that NEI might have a market advantage for their student nurses’ post-graduation, because they will be NIMART trained compared to NEIs not offering it. Nursing education institutions will also receive positive feedback from the community for having trained, qualified and experienced nurse graduates who can offer HIV services at their facilities. Moreover, this study’s findings highlighted that many nurse educators within the NEI are interested and willing to study and incorporate the NIMART course within the undergraduate nursing programme. Ngcobo and Mchunu (2019:9) agree that NEs should undergo HIV and AIDS training in order to impart knowledge of this subject to their nursing students successfully. In addition, Mboweni and Makhado’s (2020:4) study highlighted that participants wished the NIMART course could be offered within nursing colleges and universities so that they can learn how to manage TB or HIV patients before joining the Department of Health (DoH).

### Nurse Initiated Management of Antiretroviral Treatment to have recognition as a module on its own

Participants in this study accentuated the vital need for HIV and/or AIDS to be firstly recognised as a killer that is destroying our society. Given the adverse impact of HIV, participants indicated a need for the NIMART course to have its own NQF level, module hours and to have the recognition as a module on its own. In this study, participants explained that stakeholder collaboration among Department of Higher Education and Training (DHET), South African Qualification Authority (SAQA), SANC, NDoH and NEI’s is important to recognise HIV and AIDS management as a speciality. Similarly, Mboweni and Makhado (2020:10) recommended that national NDoH, nursing departments from institutions of higher learning, regional training centres, SANC, developmental partners and other relevant stakeholders, should adopt a standardised NIMART training curriculum. According to Warren et al. (2018:119), HIV specialty training is valuable and serves an important role for students committed to work in an HIV specialty setting. In addition, there could be strategic incorporation of HIV into the nursing profession curriculum, and faculty are willing to add the HIV course content when there are course materials and curriculum consultation provided (Warren et al. 2018:119). Findings in this study revealed the NIMART course should have 12 credits and 120 national module hours, including its own NQF level.

### Challenges experienced without the Nurse Initiated Management of Antiretroviral Treatment course in the undergraduate nursing programme

According to findings in this study, the NIMART course offered post-graduation leads to shortages of staff at the facilities; for example, when two professional nurses go for weeks of NIMART training, the facility becomes short staffed. Because of this shortage of staff PLWHA are left vulnerable and some return home without having ART initiated on the same day. This practise conflicts the NDoH’s (2021:20) guideline recommendations on initiating PLWHA same day on ART if there are no clinical contraindications. Another cost that emerged was that the NIMART course, when offered on completion of the undergraduate nursing programme, is more expensive than when in the undergraduate nursing programme, reason being that accommodation and meals have to be booked for weeks during the NIMART training and it is expensive. Jones and Cameroon (2017:842) stated the process of NIMART mentoring is costly and requires amounts of time and investment on important resources post-graduation.

Furthermore, the NIMART incorporation study came late, after many people had already died. Human immunodeficiency virus was first diagnosed in the 1980s; it is now 2022, and we are still talking about it, so this study is important. The study of Mboweni and Makhado (2020:6) explained that new graduate student nurses are often frustrated post-graduation because of lack of knowledge of the NIMART course and often refer patients to other facilities. However, Visser et al. (2018:5) clarified that addressing deficiency of NIMART trained nurses is central to improved service delivery among PLWHA.

### Challenges that might hinder Nurse Initiated Management of Antiretroviral Treatment incorporation into the undergraduate nursing programme

In this study, the participants explained that NIMART incorporation at undergraduate nursing programme was not feasible because of the current curriculum overload. According to the findings, the R171 programme already has too much course content. Participants also indicated that incorporating the NIMART course into the undergraduate nursing programme could require lot of time as it is going to be a tedious process and a headache. The other challenge was that with the new curriculum in place, there has been a reduction in the duration to complete the programme in colleges, from 4 to 3 years, which makes the content too much. Additionally, without stakeholder support to capacitate NEI equally, there will be a challenge to incorporate the NIMART course as the university employees are outdated with HIV and/or AIDS information and not invited to HIV conferences for capacitation. Jones and Cameron (2017:841) also indicated the NIMART course has too much content and completion is difficult post-graduation, as only 13% of nurses complete their Portfolio of Evidence (POE) and 8% receive certificates after NIMART course completion. Furthermore, it is imperative to strengthen NIMART training and explore barriers influencing the implementation for improved patient health outcomes (Mboweni & Makhado 2019:6).

## Limitations of the study

Initially, the targeted population were from a university with three campuses and a nursing college with two campuses. The limitations of this study were that in one university campus only one participant volunteered to take part in the study. Therefore, the researcher is of the opinion that findings could have been different had the participants from the other NEIs participated. Network challenges and electricity power outages restricted some participants from taking part in the interviews at agreed times. The study’s strength was its ability to conduct interviews remotely, thereby limiting the need to travel to participants for interviews.

## Recommendations

### Nursing education

This study recommends that experts in NIMART should train nurse educators so that they are able to train the undergraduate nursing students for knowledge transfer. Nursing Education Institutions will also need a curriculum committee to plan the incorporation of NIMART into the undergraduate programme, including relevant credits.

Apart from training nurse educators, participants also recommended there should be NIMART updated guidelines available on different technological platforms for easy access. The NIMART guideline will keep nurse educators up-to-date with relevant information to teach their students.

Another recommendation was for access to medication to show the students during teaching and learning. Access to internet and computer devices such as laptop was recommended in this study for students to be able to search for information regarding the NIMART course. In addition, there is a need for continuous professional development in terms of HIV and/or AIDS training for nurse educators in order for them to remain updated with information and teach updated contemporary information.

### Stakeholder collaboration

The SANC, NDoH, SAQA, NEIs and non-governmental research organisations, such as the Aurum Institute need to collaborate and give support to the NEIs on implementation of updated NIMART training and guidelines. This study also recommended that the NIMART course has the recognition as a module on its own by stakeholders.

### Ideal level to incorporate Nurse Initiated Management of Antiretroviral Treatment course

The ideal level for incorporating NIMART should be 2nd or 3rd year depending on whether the students have done anatomy, physiology, pharmacology, ethics and English modules for them to understand the concepts taught in NIMART course.

### Research

The study recommends the conducting of similar research at different provinces with larger sample size. Also recommended is that quantitative and mixed method research be used to quantify the results in future. Additionally, research funders, such as National Research Foundation (NRF), NDoH and other sponsors, should consider the prioritisation of research studies focusing on NIMART course incorporation within the undergraduate nursing programme.

## Conclusion

This study concludes that incorporation of the NIMART course into the undergraduate nursing programme is a good idea, which could benefit the student nurses, the communities and the NEIs. Nurse educators endorse the NIMART course incorporation in that it will increase student knowledge and skills to manage PLWHA in healthcare facilities, including those in rural villages, and address patient financial challenges. When the NIMART course is incorporated, new graduate nurses will immediately render HIV management services among PLWHA and curb the shortage crisis of NIMART nurses within healthcare facilities. However, the incorporation of the NIMART course faces anticipated challenges, such as current nursing programme curriculum overload and lack of stakeholder buy-in. In addition, pre-requisites, such as human and non-human resources, are necessary for a successful incorporation.
